# Supplementary material for: Pyruvate Dehydrogenase Kinase 1 inhibition mediated oxidative phosphorylation enhancement in cartilage promotes osteoarthritis progression
Source: BMC Musculoskelet Disord. 2023 Jul 20;24:597. doi: 10.1186/s12891-023-06585-6 (PMC10357736; doi:10.1186/s12891-023-06585-6)
Supplement: Supplementary file 1 — Additional file 1. [file 12891_2023_6585_MOESM1_ESM.zip › KEGG.Gsea.1653622432464/KEGG_ECM_RECEPTOR_INTERACTION.html]

Details for gene set KEGG\_ECM\_RECEPTOR\_INTERACTION[GSEA]

|  || Dataset | 55457\_collapsed\_to\_symbols.cls.cls #OA\_versus\_normal.cls.cls #OA\_versus\_normal\_repos |
| Phenotype | cls.cls#OA\_versus\_normal\_repos |
| Upregulated in class | normal |
| GeneSet | KEGG\_ECM\_RECEPTOR\_INTERACTION |
| Enrichment Score (ES) | -0.3361346 |
| Normalized Enrichment Score (NES) | -1.418199 |
| Nominal p-value | 0.014900662 |
| FDR q-value | 0.19742478 |
| FWER p-Value | 0.989 |
Table: GSEA Results Summary

  

Fig 1: Enrichment plot: KEGG\_ECM\_RECEPTOR\_INTERACTION      
 Profile of the Running ES Score & Positions of GeneSet Members on the Rank Ordered List

  

| SYMBOL | TITLE | RANK IN GENE LIST | RANK METRIC SCORE | RUNNING ES | CORE ENRICHMENT || 1 | VTN | vitronectin [Source:HGNC Symbol;Acc:HGNC:12724] | 297 | 0.753 | 0.0065 | No |
| 2 | COMP | cartilage oligomeric matrix protein [Source:HGNC Symbol;Acc:HGNC:2227] | 415 | 0.700 | 0.0249 | No |
| 3 | CHAD | chondroadherin [Source:HGNC Symbol;Acc:HGNC:1909] | 629 | 0.612 | 0.0324 | No |
| 4 | CD47 | CD47 molecule [Source:HGNC Symbol;Acc:HGNC:1682] | 1282 | 0.461 | -0.0003 | No |
| 5 | GP9 | glycoprotein IX platelet [Source:HGNC Symbol;Acc:HGNC:4444] | 1826 | 0.381 | -0.0277 | No |
| 6 | ITGA4 | integrin subunit alpha 4 [Source:HGNC Symbol;Acc:HGNC:6140] | 1854 | 0.379 | -0.0149 | No |
| 7 | COL11A1 | collagen type XI alpha 1 chain [Source:HGNC Symbol;Acc:HGNC:2186] | 2128 | 0.347 | -0.0225 | No |
| 8 | LAMC3 | laminin subunit gamma 3 [Source:HGNC Symbol;Acc:HGNC:6494] | 2177 | 0.341 | -0.0128 | No |
| 9 | THBS4 | thrombospondin 4 [Source:HGNC Symbol;Acc:HGNC:11788] | 2349 | 0.320 | -0.0135 | No |
| 10 | ITGA1 | integrin subunit alpha 1 [Source:HGNC Symbol;Acc:HGNC:6134] | 2421 | 0.311 | -0.0068 | No |
| 11 | THBS3 | thrombospondin 3 [Source:HGNC Symbol;Acc:HGNC:11787] | 2474 | 0.305 | 0.0011 | No |
| 12 | HMMR | hyaluronan mediated motility receptor [Source:HGNC Symbol;Acc:HGNC:5012] | 2634 | 0.287 | 0.0000 | No |
| 13 | SV2B | synaptic vesicle glycoprotein 2B [Source:HGNC Symbol;Acc:HGNC:16874] | 2719 | 0.278 | 0.0044 | No |
| 14 | ITGB5 | integrin subunit beta 5 [Source:HGNC Symbol;Acc:HGNC:6160] | 2823 | 0.267 | 0.0069 | No |
| 15 | SDC2 | syndecan 2 [Source:HGNC Symbol;Acc:HGNC:10659] | 2880 | 0.262 | 0.0129 | No |
| 16 | SV2C | synaptic vesicle glycoprotein 2C [Source:HGNC Symbol;Acc:HGNC:30670] | 2998 | 0.250 | 0.0136 | No |
| 17 | DAG1 | dystroglycan 1 [Source:HGNC Symbol;Acc:HGNC:2666] | 3203 | 0.230 | 0.0067 | No |
| 18 | COL4A4 | collagen type IV alpha 4 chain [Source:HGNC Symbol;Acc:HGNC:2206] | 3592 | 0.195 | -0.0159 | No |
| 19 | LAMB3 | laminin subunit beta 3 [Source:HGNC Symbol;Acc:HGNC:6490] | 3679 | 0.188 | -0.0152 | No |
| 20 | SPP1 | secreted phosphoprotein 1 [Source:HGNC Symbol;Acc:HGNC:11255] | 4205 | 0.137 | -0.0507 | No |
| 21 | TNC | tenascin C [Source:HGNC Symbol;Acc:HGNC:5318] | 4874 | 0.082 | -0.0996 | No |
| 22 | THBS2 | thrombospondin 2 [Source:HGNC Symbol;Acc:HGNC:11786] | 4943 | 0.077 | -0.1019 | No |
| 23 | LAMB4 | laminin subunit beta 4 [Source:HGNC Symbol;Acc:HGNC:6491] | 5111 | 0.063 | -0.1125 | No |
| 24 | SDC3 | syndecan 3 [Source:HGNC Symbol;Acc:HGNC:10660] | 5114 | 0.063 | -0.1101 | No |
| 25 | COL5A2 | collagen type V alpha 2 chain [Source:HGNC Symbol;Acc:HGNC:2210] | 5262 | 0.051 | -0.1196 | No |
| 26 | SV2A | synaptic vesicle glycoprotein 2A [Source:HGNC Symbol;Acc:HGNC:20566] | 5398 | 0.040 | -0.1285 | No |
| 27 | COL5A1 | collagen type V alpha 1 chain [Source:HGNC Symbol;Acc:HGNC:2209] | 5414 | 0.039 | -0.1282 | No |
| 28 | GP1BB | glycoprotein Ib platelet subunit beta [Source:HGNC Symbol;Acc:HGNC:4440] | 5457 | 0.037 | -0.1300 | No |
| 29 | LAMA4 | laminin subunit alpha 4 [Source:HGNC Symbol;Acc:HGNC:6484] | 5473 | 0.035 | -0.1298 | No |
| 30 | SDC1 | syndecan 1 [Source:HGNC Symbol;Acc:HGNC:10658] | 5526 | 0.031 | -0.1327 | No |
| 31 | RELN | reelin [Source:HGNC Symbol;Acc:HGNC:9957] | 5635 | 0.021 | -0.1402 | No |
| 32 | COL4A6 | collagen type IV alpha 6 chain [Source:HGNC Symbol;Acc:HGNC:2208] | 5975 | -0.003 | -0.1666 | No |
| 33 | AGRN | agrin [Source:HGNC Symbol;Acc:HGNC:329] | 6155 | -0.017 | -0.1799 | No |
| 34 | GP1BA | glycoprotein Ib platelet subunit alpha [Source:HGNC Symbol;Acc:HGNC:4439] | 6991 | -0.079 | -0.2419 | No |
| 35 | ITGAV | integrin subunit alpha V [Source:HGNC Symbol;Acc:HGNC:6150] | 7067 | -0.085 | -0.2444 | No |
| 36 | TNXB | tenascin XB [Source:HGNC Symbol;Acc:HGNC:11976] | 7189 | -0.095 | -0.2501 | No |
| 37 | LAMA2 | laminin subunit alpha 2 [Source:HGNC Symbol;Acc:HGNC:6482] | 7392 | -0.110 | -0.2615 | No |
| 38 | ITGA9 | integrin subunit alpha 9 [Source:HGNC Symbol;Acc:HGNC:6145] | 7785 | -0.140 | -0.2865 | No |
| 39 | LAMB2 | laminin subunit beta 2 [Source:HGNC Symbol;Acc:HGNC:6487] | 7841 | -0.144 | -0.2852 | No |
| 40 | ITGA2 | integrin subunit alpha 2 [Source:HGNC Symbol;Acc:HGNC:6137] | 7857 | -0.145 | -0.2806 | No |
| 41 | ITGB7 | integrin subunit beta 7 [Source:HGNC Symbol;Acc:HGNC:6162] | 7873 | -0.146 | -0.2761 | No |
| 42 | CD36 | CD36 molecule [Source:HGNC Symbol;Acc:HGNC:1663] | 7985 | -0.154 | -0.2787 | No |
| 43 | COL1A1 | collagen type I alpha 1 chain [Source:HGNC Symbol;Acc:HGNC:2197] | 8133 | -0.165 | -0.2836 | No |
| 44 | GP5 | glycoprotein V platelet [Source:HGNC Symbol;Acc:HGNC:4443] | 8215 | -0.173 | -0.2831 | No |
| 45 | TNR | tenascin R [Source:HGNC Symbol;Acc:HGNC:11953] | 8715 | -0.210 | -0.3138 | No |
| 46 | COL3A1 | collagen type III alpha 1 chain [Source:HGNC Symbol;Acc:HGNC:2201] | 8784 | -0.215 | -0.3106 | No |
| 47 | ITGA6 | integrin subunit alpha 6 [Source:HGNC Symbol;Acc:HGNC:6142] | 9112 | -0.240 | -0.3267 | Yes |
| 48 | VWF | von Willebrand factor [Source:HGNC Symbol;Acc:HGNC:12726] | 9180 | -0.246 | -0.3222 | Yes |
| 49 | ITGA10 | integrin subunit alpha 10 [Source:HGNC Symbol;Acc:HGNC:6135] | 9296 | -0.256 | -0.3211 | Yes |
| 50 | ITGB8 | integrin subunit beta 8 [Source:HGNC Symbol;Acc:HGNC:6163] | 9442 | -0.268 | -0.3219 | Yes |
| 51 | HSPG2 | heparan sulfate proteoglycan 2 [Source:HGNC Symbol;Acc:HGNC:5273] | 9449 | -0.269 | -0.3118 | Yes |
| 52 | ITGB6 | integrin subunit beta 6 [Source:HGNC Symbol;Acc:HGNC:6161] | 9614 | -0.284 | -0.3134 | Yes |
| 53 | ITGA7 | integrin subunit alpha 7 [Source:HGNC Symbol;Acc:HGNC:6143] | 9724 | -0.294 | -0.3103 | Yes |
| 54 | COL11A2 | collagen type XI alpha 2 chain [Source:HGNC Symbol;Acc:HGNC:2187] | 9734 | -0.295 | -0.2994 | Yes |
| 55 | COL1A2 | collagen type I alpha 2 chain [Source:HGNC Symbol;Acc:HGNC:2198] | 9985 | -0.320 | -0.3063 | Yes |
| 56 | COL2A1 | collagen type II alpha 1 chain [Source:HGNC Symbol;Acc:HGNC:2200] | 10122 | -0.333 | -0.3038 | Yes |
| 57 | ITGB4 | integrin subunit beta 4 [Source:HGNC Symbol;Acc:HGNC:6158] | 10159 | -0.337 | -0.2933 | Yes |
| 58 | ITGB3 | integrin subunit beta 3 [Source:HGNC Symbol;Acc:HGNC:6156] | 10310 | -0.352 | -0.2912 | Yes |
| 59 | IBSP | integrin binding sialoprotein [Source:HGNC Symbol;Acc:HGNC:5341] | 10351 | -0.356 | -0.2803 | Yes |
| 60 | COL6A2 | collagen type VI alpha 2 chain [Source:HGNC Symbol;Acc:HGNC:2212] | 10385 | -0.359 | -0.2687 | Yes |
| 61 | COL5A3 | collagen type V alpha 3 chain [Source:HGNC Symbol;Acc:HGNC:14864] | 10456 | -0.368 | -0.2597 | Yes |
| 62 | LAMC2 | laminin subunit gamma 2 [Source:HGNC Symbol;Acc:HGNC:6493] | 10531 | -0.376 | -0.2506 | Yes |
| 63 | LAMC1 | laminin subunit gamma 1 [Source:HGNC Symbol;Acc:HGNC:6492] | 10642 | -0.387 | -0.2439 | Yes |
| 64 | TNN | tenascin N [Source:HGNC Symbol;Acc:HGNC:22942] | 10713 | -0.395 | -0.2339 | Yes |
| 65 | ITGA8 | integrin subunit alpha 8 [Source:HGNC Symbol;Acc:HGNC:6144] | 11046 | -0.437 | -0.2426 | Yes |
| 66 | LAMB1 | laminin subunit beta 1 [Source:HGNC Symbol;Acc:HGNC:6486] | 11135 | -0.448 | -0.2318 | Yes |
| 67 | COL6A1 | collagen type VI alpha 1 chain [Source:HGNC Symbol;Acc:HGNC:2211] | 11204 | -0.457 | -0.2191 | Yes |
| 68 | GP6 | glycoprotein VI platelet [Source:HGNC Symbol;Acc:HGNC:14388] | 11341 | -0.477 | -0.2109 | Yes |
| 69 | CD44 | CD44 molecule (Indian blood group) [Source:HGNC Symbol;Acc:HGNC:1681] | 11439 | -0.494 | -0.1990 | Yes |
| 70 | THBS1 | thrombospondin 1 [Source:HGNC Symbol;Acc:HGNC:11785] | 11498 | -0.501 | -0.1838 | Yes |
| 71 | ITGA2B | integrin subunit alpha 2b [Source:HGNC Symbol;Acc:HGNC:6138] | 11645 | -0.524 | -0.1746 | Yes |
| 72 | COL4A2 | collagen type IV alpha 2 chain [Source:HGNC Symbol;Acc:HGNC:2203] | 11940 | -0.580 | -0.1747 | Yes |
| 73 | SDC4 | syndecan 4 [Source:HGNC Symbol;Acc:HGNC:10661] | 12038 | -0.603 | -0.1585 | Yes |
| 74 | LAMA3 | laminin subunit alpha 3 [Source:HGNC Symbol;Acc:HGNC:6483] | 12449 | -0.724 | -0.1620 | Yes |
| 75 | ITGA5 | integrin subunit alpha 5 [Source:HGNC Symbol;Acc:HGNC:6141] | 12474 | -0.737 | -0.1349 | Yes |
| 76 | COL6A3 | collagen type VI alpha 3 chain [Source:HGNC Symbol;Acc:HGNC:2213] | 12506 | -0.750 | -0.1078 | Yes |
| 77 | ITGB1 | integrin subunit beta 1 [Source:HGNC Symbol;Acc:HGNC:6153] | 12566 | -0.780 | -0.0817 | Yes |
| 78 | LAMA5 | laminin subunit alpha 5 [Source:HGNC Symbol;Acc:HGNC:6485] | 12692 | -0.859 | -0.0576 | Yes |
| 79 | COL4A1 | collagen type IV alpha 1 chain [Source:HGNC Symbol;Acc:HGNC:2202] | 12703 | -0.866 | -0.0243 | Yes |
| 80 | FN1 | fibronectin 1 [Source:HGNC Symbol;Acc:HGNC:3778] | 12820 | -1.005 | 0.0062 | Yes |
Table: GSEA details [plain text format]

  

Fig 2: KEGG\_ECM\_RECEPTOR\_INTERACTION      
 Blue-Pink O' Gram in the Space of the Analyzed GeneSet

  

Fig 3: KEGG\_ECM\_RECEPTOR\_INTERACTION: Random ES distribution      
 Gene set null distribution of ES for **KEGG\_ECM\_RECEPTOR\_INTERACTION**
